# Reinforcement learning method for machining deformation control based on meta-invariant feature space

**DOI:** 10.1186/s42492-022-00123-2

**Published:** 2022-11-24

**Authors:** Yujie Zhao, Changqing Liu, Zhiwei Zhao, Kai Tang, Dong He

**Affiliations:** 1 College of Mechanical and Electrical Engineering/National Key Laboratory of Science and Technology on Helicopter Transmission, Nanjing University of Aeronautics and Astronautics, Nanjing, 210016 China; 2 The Smart Manufacturing Thrust, Hong Kong University of Science and Technology (GZ), Guangzhou, 511458 China

**Keywords:** Machining deformation, Residual stress, Deformation control, Meta-invariant feature space; Reinforcement learning

## Abstract

Precise control of machining deformation is crucial for improving the manufacturing quality of structural aerospace components. In the machining process, different batches of blanks have different residual stress distributions, which pose a significant challenge to machining deformation control. In this study, a reinforcement learning method for machining deformation control based on a meta-invariant feature space was developed. The proposed method uses a reinforcement-learning model to dynamically control the machining process by monitoring the deformation force. Moreover, combined with a meta-invariant feature space, the proposed method learns the internal relationship of the deformation control approaches under different stress distributions to achieve the machining deformation control of different batches of blanks. Finally, the experimental results show that the proposed method achieves better deformation control than the two existing benchmarking methods.

## Introduction

Structural aerospace components are pivotal components of an aircraft. They are subjected to strict manufacturing standards to ensure improved assembly quality, service performance, product life, and other critical performance criteria. However, because of the high material removal rate during machining of structural components, their large size, and complex residual stress distributions, severe deformation often occurs during their production and processing, for example, bending, twisting, or their combination [1]. The European Union spends 10 million euros yearly on the aerospace manufacturing industry to cope with machining deformation problems [2]. Therefore, controlling the machining deformation of structural aerospace components is a critical and challenging problem in aviation manufacturing.

**Fig. 1:**
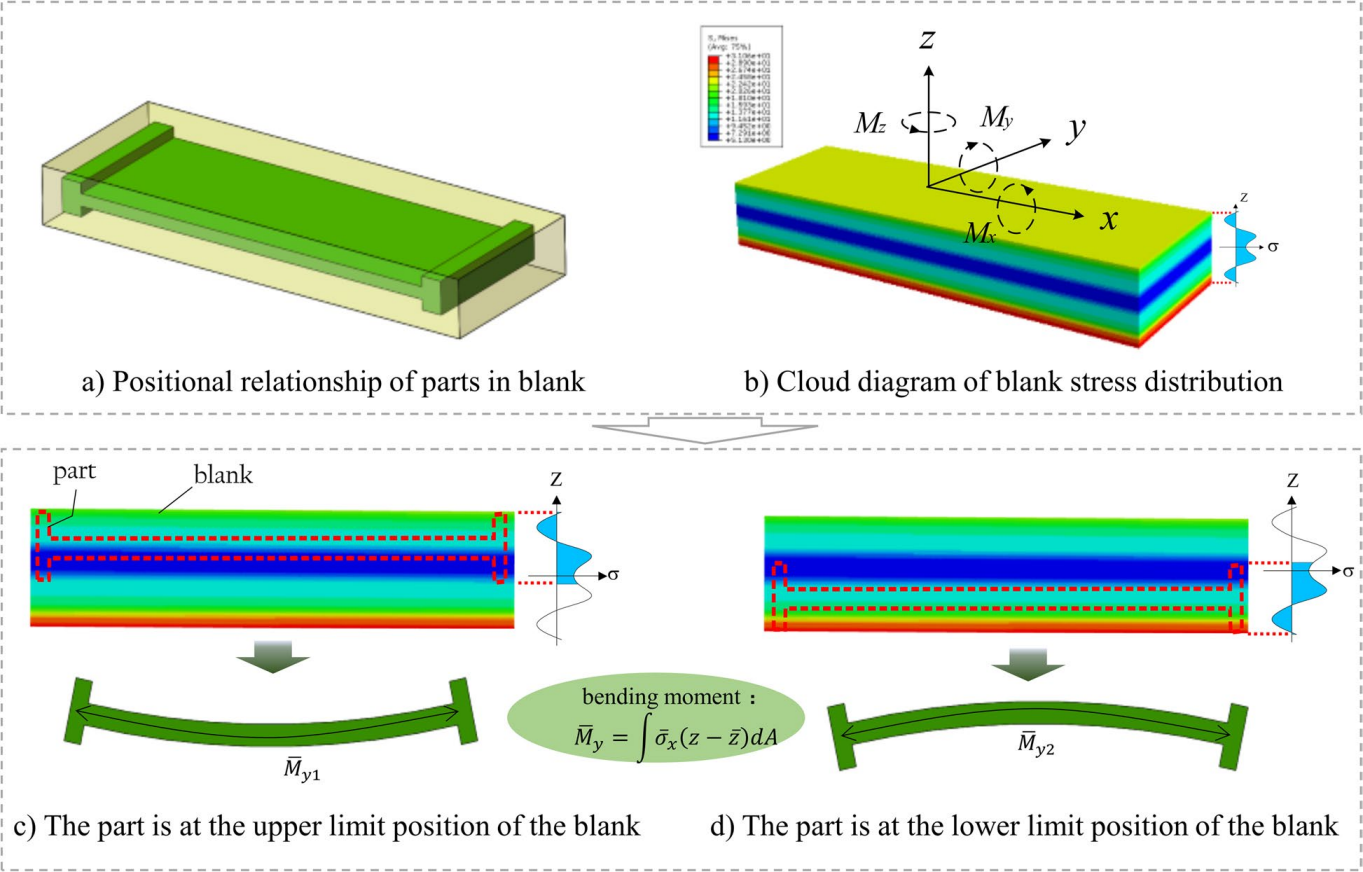
Influence of machining position on deformation of machined part

The deformation control of structural components for machining is shown in Fig. 1. Given a raw blank enclosing the design part, the machining process removes the material between them (Fig. 1a). Because of the initial residual stress distribution in the blank (Fig. 1b), the machined part deforms. In particular, the degree of deformation is determined by the stress distribution of the blank and the relative position of the part within the blank [3], as shown in Figs. 1c and d. Nevertheless, the dimensions of the blank are larger than those of the part, providing a space for position adjustment. Therefore, given a specific initial residual stress distribution of a blank, deformation control requires adjusting the part position in the blank to minimize the resultant deformation of the machined part.

Although the residual stress distribution of a blank can be obtained to predict and control the part deformation, it is challenging to determine a general optimal machining positioning approach tending to a general blank. This is because the stress distribution varies significantly among blanks owing to different heat treatment and prestretching parameters and the random error. Despite the development of traditional machining deformation control methods based on analytical and numerical modeling, these methods significantly depend on the prior residual stress and constitutive equations, both of which are difficult to obtain; thus, they cannot guarantee deformation control accuracy. As emerging data-acquisition techniques can collect a large amount of actual data during machining, it has become possible to develop data-driven approaches for solving manufacturing problems [4], such as industrial robot grasping based on deep reinforcement learning [5], remaining service life prediction of machinery based on deep learning [6], and tool wear prediction based on causal inference [7]. Nevertheless, the significant variations in blank materials and machining conditions make it challenging to adapt many existing data-driven methods to a specific machining deformation control problem.

To address these problems, the authors propose a reinforcement learning method for machining deformation control based on the meta-invariant feature space, which is a method for cross-condition learning based on meta-learning. Instead of measuring the stress distributions in the entire blank, the deformation force [8, 9] is determined on a few monitoring points as the input. This approach reduces the problem complexity and fully utilizes the nonlinear mapping of machine learning between the force input and adjustment position output. A model can be established using a meta-invariant feature space by learning the underlying laws under different stress distributions. This enables the model to dynamically adjust the machining allowance and determine the final machining position based on the online-monitored machining data. Before proceeding to the methodology, related work is reviewed first.

### Related work

Machining deformation is an essential factor that affects the quality of parts, and its effective control has been investigated extensively. Relevant existing methods are divided into two categories: mechanism-based and data-driven.

#### Mechanism-based methods

In mechanism-based methods for deformation control, the residual stress of the material to be machined is measured first, and machining deformation is then predicted by analytical or numerical modeling to adjust the process based on the predicted results. Wang et al. [10] used finite element software to simulate the removal process of aluminum alloys and reported that the release and redistribution of residual stress is the main cause of machining deformation. Cerutti and Mocellin [11] considered the effect of the initial residual stress and used a numerical tool to analyze the effect of the machining sequence on machining deformation. Wang et al. [12] developed a slope method to measure the residual stress of materials and controlled the deformation by optimizing the machining position. Wang et al. [13] established an analytical model for predicting the machining deformation of multi-frame components based on an energy method and minimized the deformation by optimizing the cutting parameters using a cornstarch suspension. Li et al. [14] established a deformation prediction model between the initial residual stress and finishing allowance. They developed a linear-programming optimization model to optimize the overall machining deformation. Jiang et al. [15] proposed a nonuniform allowance allocation method based on the interim state stiffness of machining features. Their proposed method can effectively improve the stiffness of a part and hence, reduce its deformation.

The accuracy of mechanism-based deformation control significantly depends on the accuracy of the residual stress measurements. Current methods for measuring residual stress include destructive and nondestructive test methods [16], but their accuracy and efficiency do not satisfy the high requirements of deformation prediction and control.

#### Data-driven methods

In the data-driven manufacturing era, the extensive growth of data has completely changed data collection and analysis methods [17]. Process control based on data monitoring during machining processes has gradually become effective for improving machining quality [18]. Bakker et al. [19] proposed a new fixture design concept in which the clamping force is controlled by combining sensors and active clamping elements to minimize the deformation of parts during manufacturing. Li et al. [20] developed responsive fixtures for monitoring and controlling machining deformation. Hao et al. [21] reduced machining deformation by controlling the machining sequence, pre-deformation [22], and machining allowance allocation [23]. Gonzalo et al. [24] developed an intelligent fixture to correct the machining deformation of parts by evaluating the reaction force of clamping points. However, as the machining deformation of parts is highly nonlinear with respect to the observed data, it is difficult to satisfy the accuracy and reliability requirements of deformation control by relying only on the monitoring data in current machining processes.

With the rapid development of automation, an increasing number of tasks have relied on artificial intelligence applications [25]. Many machine-learning methods have been developed to characterize the nonlinearity of deformation control. Reinforcement learning algorithms such as deep Q-networks (DQNs) [26] have attracted attention in industrial control systems [27], path planning [28], manufacturing scheduling [29], and other industries because of their excellent learning ability. Recently, a reinforcement learning algorithm has been applied to deformation control by dynamically selecting machining processes using monitored machining data [30]; however, its generalization to new problems is somewhat limited. To this end, transfer learning [31] applies learned knowledge to new problems. For example, Alam et al. [32] and Liu et al. [33] used the transfer learning method to enable data-driven models to exhibit improved adaptability in learning the parameters of a manufacturing process and drilling-burr prediction, respectively. However, transfer learning is not always effective when significant differences exist between tasks. Meta-learning [34] is a learn-to-learn algorithm that shows satisfactory results in generalization to new tasks. Liu et al. [35] proposed a meta-invariant feature space method to accurately predict tool wear across conditions with only a few new samples. Li et al. [36] developed a multitask reinforcement learning method combined with meta-learning that could enable an unmanned aerial vehicle to adapt to a new target motion mode faster with only a few training steps. Xiao et al. [37] used a meta-reinforcement learning algorithm to determine optimal machining parameters during turning. Liu et al. [38] proposed a meta-reinforcement learning method that incorporated simulations with actual data for machining deformation control of the finishing machining process.

Inspired by the meta-learning method, a reinforcement learning method combined with a meta-invariant feature space is proposed in this study. The proposed method has distinct advantages over existing methods: (1) Two subnetworks are established for the model to learn the invariant features of the paired conditions; (2) An autoencoder is incorporated into the model, which can map the input to latent variables as invariant features; (3) Reinforcement learning is incorporated into the model, which can dynamically control the machining positions; and (4) The meta-model can learn the underlying and intrinsic features under different stress distributions and control the machining deformation of different blanks.

## Methods

**Fig. 2:**
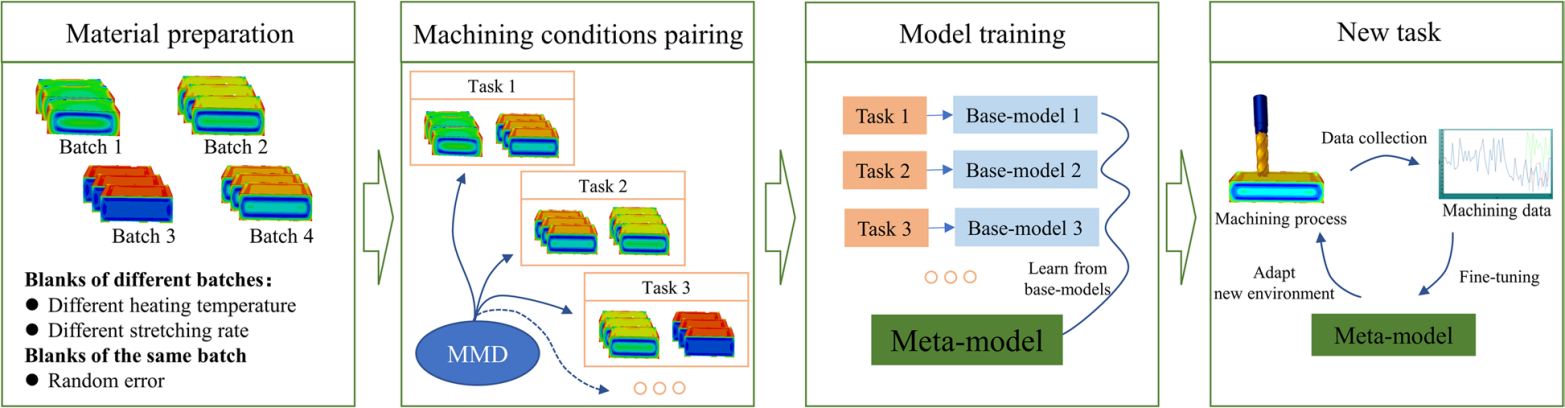
Flowchart of proposed method

As mentioned in the introduction, the residual stress distributions in different batches of blanks are different owing to the variation in blank material preparation, such as the heat treatment parameters and prestretching. In addition, perturbations of stress distributions occur in the same batch of blanks owing to random errors. In this study, these two reasons for distribution variations were considered. First, different groups for different material preparation parameters were set, and random perturbations within each group were added to reflect the random errors within each batch. Next, the deformation forces on a few monitored points were selected as the input, and different batches were paired, that is, machining conditions, using the maximum mean discrepancy (MMD) method. For each pair, a base-model consisting of two subnetworks was then established to learn its invariant features. The learned model was combined with the principle of meta-invariant feature space [35] to make the model learn the intrinsic relationship guiding different batch control approaches to achieve stable and accurate decision-making. Finally, when facing a new machining task, the meta-model will use a small amount of monitoring data to fine-tune the model parameters to adapt to new tasks and achieve accurate machining deformation control. The flowchart of the proposed method is shown in Fig. 2.

**Fig. 3:**
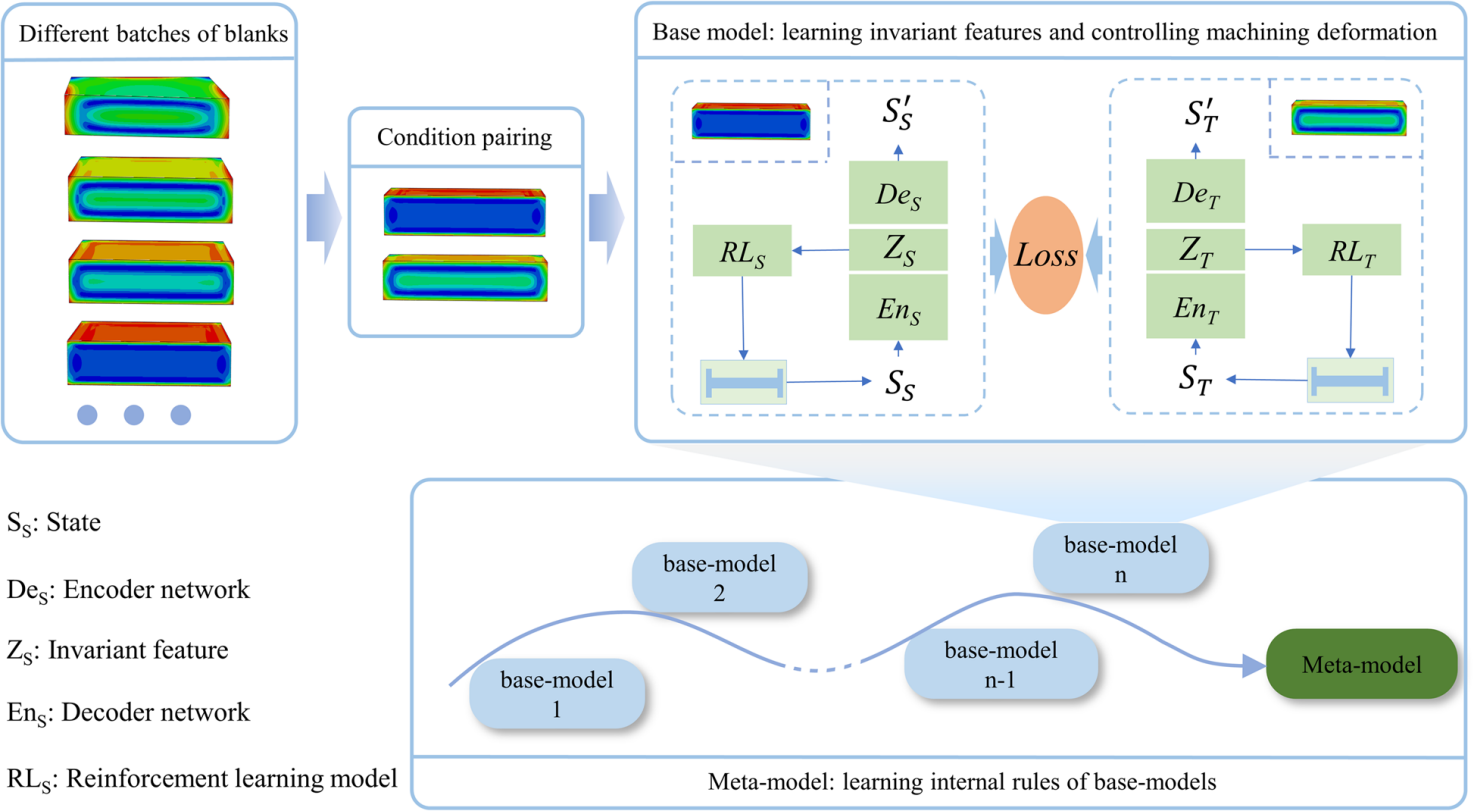
Learning framework of reinforcement learning method for machining position adjustment strategy based on meta-invariant feature space

The algorithm framework consists of base-models and a meta-model (Fig. 3). Each base-model learns a specific task, making process decisions according to a specific pair of machining conditions. First, the groups are paired. For each pair (*S*, *T*), a base-model is defined. Next, cooperative learning is applied to map the marginal distributions of *S* and *T* into an invariant feature space of the base-model, thereby closing the marginal distributions for different conditions. The base-model then passes the learned results to a meta-model. Finally, the meta-model learns more helpful information in related tasks from the obtained base-models to attain a meta-invariant feature space. In summary, the entire algorithm includes three aspects: condition pairing, base-model learning, and meta-model learning. Each aspect is described below.

### Machining condition pairing

Before training the meta-invariant feature space model, the first step is to pair the machining conditions. In this study, MMD, the most widely used in marginal distribution adaptation, was used to measure the distance of the margin distribution between the two conditions. Specifically, for condition set ${\left\{{\mathcal{C}}_{n}\right\}}_{n=1,\dots ,N}$ under *N* conditions, the first condition, ${\mathcal{C}}_{1}$, is selected as the current condition ${\mathcal{C}}_{\mathrm{cur}}={\mathcal{C}}_{1}$, and the MMDs between ${\mathcal{C}}_{\mathrm{cur}}$ and the remaining *N*-1 candidate conditions are calculated. The candidate condition, ${\mathcal{C}}_{\mathrm{can}}$, which has the minimum MMD to ${\mathcal{C}}_{\mathrm{cur}}$, is paired with the current condition, for example, $\left({\mathcal{C}}_{\mathrm{cur}},{\mathcal{C}}_{\mathrm{can}}\right)$. Next, ${\mathcal{C}}_{\mathrm{cur}}$ is replaced by the just paired ${\mathcal{C}}_{\mathrm{can}}$, and the new ${\mathcal{C}}_{\mathrm{cur}}$ is paired with the remaining *N*-2 candidate conditions. This procedure is repeated until all conditions are paired.

The *S* and *T* data samples are embedded into the reproduced kernel Hilbert space ${\mathcal{H}}^{Z}$ to calculate the MMDs of both conditions, in which each function $f$ corresponds to a feature map. Let ${P}^{T}$ and ${P}^{S}$ denote the data distributions in *S* and *T*, respectively. The means of the data distributions, that is, ${\mu }_{{P}^{S}}$ and ${\mu }_{{P}^{T}}$, are embedded under $f$ as follows.

1 $${\mu }_{{P}^{con}}\in {\mathcal{H}}^{Z} \mathrm{s}.\mathrm{t}. {E}_{x}\left[f\right]={\langle f,{\mu }_{{P}^{con}}\rangle }_{\mathcal{H}}, \forall f\in {\mathcal{H}}^{Z}, con=S, T$$

Equation (1) represents the kernel concept that simplifies the calculation of the feature space transformation. The square of the MMD between ${P}^{T}$ and ${P}^{S}$ can thus, be expressed as follows:

2 $$\begin{array}{c}{MMD}^{2}\left({P}^{S},{P}^{T};\mathcal{F}\right)={\left[\mathrm{sup}\left({E}_{x}\left[f\left({x}^{S}\right)\right]-{E}_{x}\left[f\left({x}^{T}\right)\right]\right)\right]}^{2}\\\\ ={\Vert {\mu }_{{P}^{S}}-{\mu }_{{P}^{T}}\Vert }_{\mathcal{H}}^{2}\\\\ \begin{array}{l}={E}_{{x}_{i}^{S},{x}_{j}^{S}}\left[k\left({x}_{i}^{S},{x}_{j}^{S}\right)\right]-2{E}_{{x}_{i}^{S},{x}_{j}^{T}}\left[k\left({x}_{i}^{S},{x}_{j}^{T}\right)\right]+{E}_{{x}_{i}^{T},{x}_{j}^{T}}\left[k\left({x}_{i}^{T},{x}_{j}^{T}\right)\right]\\\\ =\frac{1}{{m}^{2}}\sum_{i,j=1}^{m}k\left({x}_{i}^{S},{x}_{j}^{S}\right)-\frac{2}{mn}\sum_{i,j=1}^{m,n}k\left({x}_{i}^{S},{x}_{j}^{T}\right)+\frac{1}{{n}^{2}}\sum_{i,j=1}^{n}k\left({x}_{i}^{T},{x}_{j}^{T}\right)\end{array}\end{array}$$

where ${x}_{i}^{S}$ is the *i*^th^ sample from condition *S*, which is a vector, $k\left({x}_{i}^{con},{x}_{j}^{con}\right)=\mathrm{exp}\left(-\frac{{\Vert {x}_{i}^{con}-{x}_{j}^{con}\Vert }^{2}}{2{\sigma }^{2}}\right)$ represents the Gaussian kernel, $\mathcal{F}$ is the function space of *f*, and *m* and *n* are the numbers of samples in *S* and *T*, respectively.

### Base-model learning

**Fig. 4:**
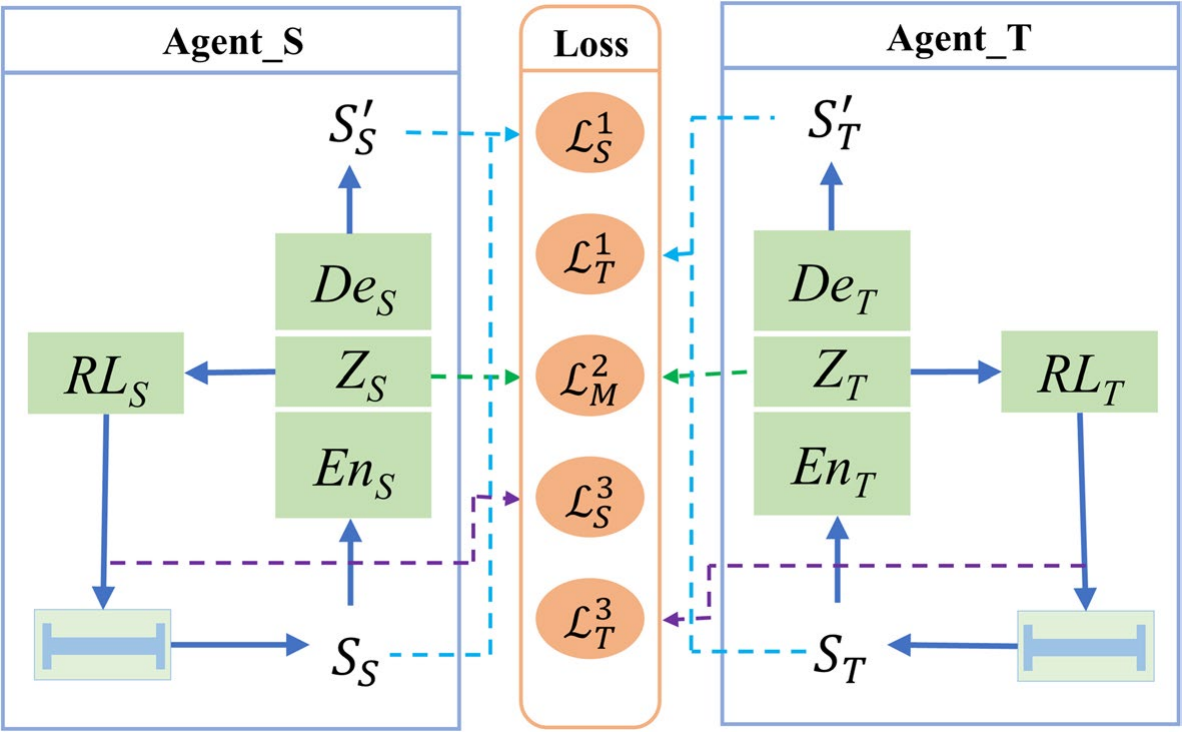
Base model with two subnetworks. Each subnetwork has an autoencoder and a reinforcement learning component

The monitoring data are somewhat different because of the different residual-stress distributions from different batches of blanks. The model trained only in a specific batch could not achieve an ideal deformation control effect in other batches. Therefore, an invariant feature space [39] was designed in this study, into which the features under different distributions were transformed through collaborative learning of the reinforcement learning model under paired machining conditions. Therefore, common features under different machining conditions were extracted to lay a foundation for meta-learning to determine the intrinsic laws of the model. The invariant feature space model framework is shown in Fig. 4, and the parameters are defined as follows:

1. ${S}_{S}$ and ${S}_{T}$ are the monitoring deformation force data of Agent_S and Agent_T, respectively;
2. ${En}_{S}$ and ${En}_{T}$ are the encoding networks of Agent_S and Agent_T, respectively;
3. ${Z}_{S}$ and ${Z}_{T}$ are the latent variables of Agent_S and Agent_T, respectively;
4. ${De}_{S}$ and ${De}_{T}$ are the decoding networks of Agent_S and Agent_T, respectively;
5. ${RL}_{S}$ and ${RL}_{T}$ are the reinforcement learning networks used for process decision-making of Agent_S and Agent_T, respectively;
6. ${\mathcal{L}}_{S}^{1}$ and ${\mathcal{L}}_{T}^{1}$ are the reconstruction losses of monitoring data ${S}_{S}$ and ${S}_{T}$, respectively;
7. ${\mathcal{L}}_{M}^{2}$ is the match loss of the latent variables ${Z}_{S}$ and ${Z}_{T}$;
8. ${\mathcal{L}}_{S}^{3}$ and ${\mathcal{L}}_{T}^{3}$ are the losses of reinforcement learning models ${RL}_{S}$ and ${RL}_{T}$, respectively.

The base-model uses Agent_S and Agent_T to take machining decisions on conditions *S* and *T*, respectively. First, states ${S}_{S}$ and ${S}_{T}$ are mapped onto latent variables ${Z}_{S}$ and ${Z}_{T}$ to construct the invariant feature space through encoding networks ${En}_{S}$ and ${En}_{T}$, respectively. Simultaneously, decoding networks ${De}_{S}$ and ${De}_{T}$ are trained, forming an autoencoder whose outputs are ${S}_{S}^{^{\prime}}$ and ${S}_{T}^{^{\prime}}$, respectively, to ensure the reversibility of the mapping, that is, to retain the information of the input data as much as possible. The latent variables of the two autoencoders, ${Z}_{S}$ and ${Z}_{T}$, are used to train the invariant feature space of the pair (*S*, *T*). The reinforcement learning models, ${RL}_{S}$ and ${RL}_{T}$, then determine the machining processes according to latent variables ${Z}_{S}$ and ${Z}_{T}$. In base-model learning, the loss function of the base-model comprises three parts: reconstruction, match, and reinforcement learning losses.

1. Reconstruction loss:
  3 $${\mathcal{L}}_{S}^{1}=MSE({S}_{S}-{S}_{S}^{^{\prime}})$$
  4 $${\mathcal{L}}_{T}^{1}=MSE({S}_{T}-{S}_{T}^{^{\prime}})$$
  Here, *MSE* denotes the mean square error.
2. Match loss:
  5 $${\mathcal{L}}_{M}^{2}=\frac{1}{\left|{z}^{S}\right|}\sum l({Z}_{S},{Z}_{T})$$
  In Eq. (5), $l\left({Z}_{S},{Z}_{T}\right)=1-\mathrm{cos}\left({Z}_{S},{Z}_{T}\right)=\frac{{\Vert {Z}_{S}\Vert }_{2}\bullet {\Vert {Z}_{T}\Vert }_{2}-{Z}_{S}\bullet {Z}_{T}}{{\Vert {Z}_{S}\Vert }_{2}\bullet {\Vert {Z}_{T}\Vert }_{2}}$. The cosine distance is adopted as the metric distance between the latent variables rather than the absolute difference in length because the angular difference can reflect the characteristics of the encoded monitoring signal more effectively.
3. Reinforcement learning loss:

The reinforcement learning model for each condition was trained based on monitoring data and latent variables. During the machining process, the model determined the machining position to obtain the final part. In terms of implementation, the DQN algorithm was used in this study to achieve machining deformation control, in which the state, action, and reward are indispensable parts of the algorithm.

#### State

**Fig. 5:**
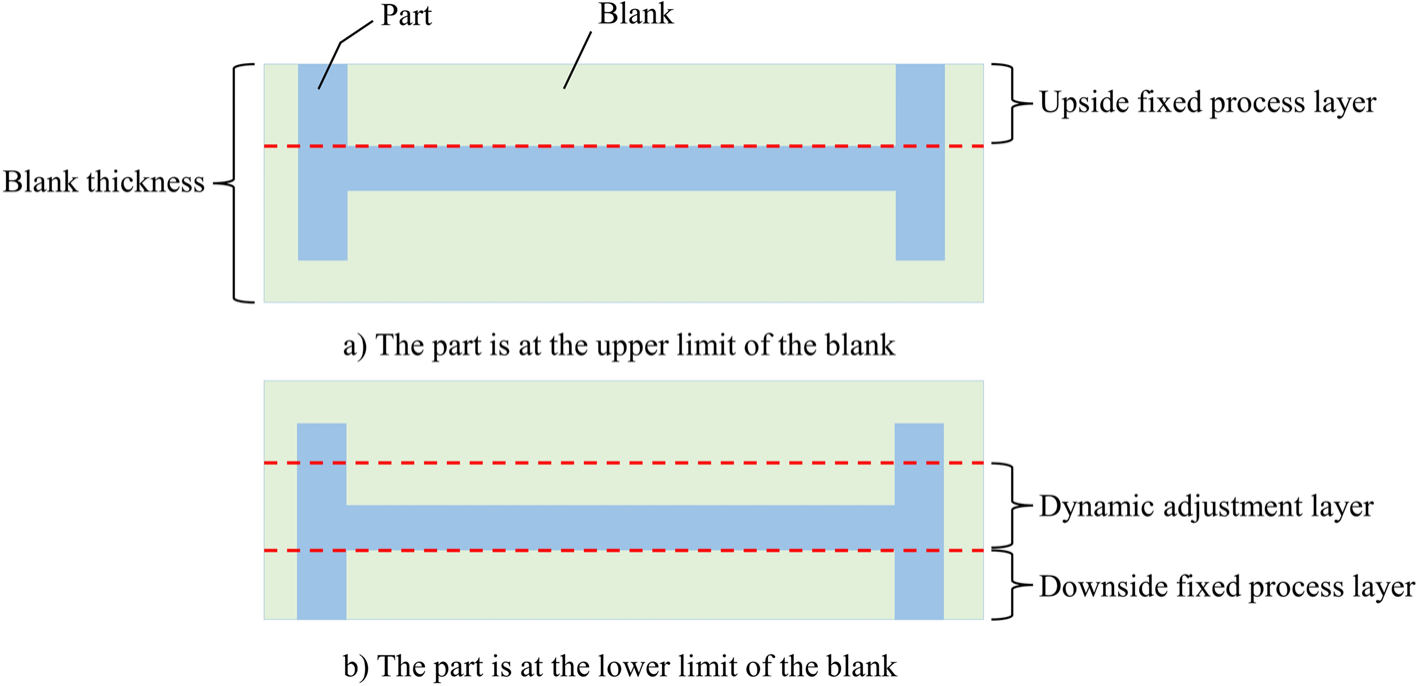
The layering diagram of a blank

The deformation force can be monitored in real time during machining and contains deformation and stress information of the parts [8, 9]. By taking the position adjustment of a part as an example, the blank can be divided into two fixed process layers and one dynamic adjustment layer before machining [23], as shown in Fig. 5. The cavities of the fixed process layers are removed by multilayer rough machining, during which the deformation force information can reflect the initial stress information of the blank. After machining the fixed process layers, the dynamic adjustment layer is machined, during which the current machining position and deformation force data represent the current machining state. Therefore, the state of reinforcement learning is a combination of (1) the deformation force of the fixed process layer, (2) current machining position of the dynamic adjustment layer, and (3) deformation force of the dynamic adjustment layer.

#### Action

The dynamic adjustment layer is divided into several sublayers with specific intervals; that is, several machining positions are determined, and each is regarded as an action.

#### Reward

The reward function represents the direction of training optimization. Because the deformation of the machined part increases with increasing deformation force, a low force is required for deformation control. Therefore, the negative maximum absolute value of the deformation force is considered the reward function for reinforcement learning:

6 $$reward=-\underset{n}{\mathrm{max}}\left|{F}_{n}\right|$$

where ${F}_{n}$ is the deformation force of the *n*^th^ monitoring point during the machining process. When the deformation force is large, the reward is small; thus, the model reduces the possibility of selecting this position.

The DQN model has two value functions with the same structure, but different parameters expressed as *target_net* and *eval_net*. *eval_net* is used to evaluate the greedy policy, whereas *target_net* is used to estimate its value. Therefore, based on the parameter-updating mechanism of the DQN model, the loss functions ${\mathcal{L}}_{S}^{3}$ and ${\mathcal{L}}_{T}^{3}$ of Agent_S and Agent_T can be expressed as follows, respectively:

7 $${\mathcal{L}}_{S}^{3}={[{reward}_{S}+\gamma *\mathrm{max}{Q}_{S}^{target}-{Q}_{S}^{eval}]}^{2}$$

8 $${\mathcal{L}}_{T}^{3}={[{reward}_{T}+\gamma *\mathrm{max}{Q}_{T}^{target}-{Q}_{T}^{eval}]}^{2}$$

where *γ* is a discount factor, ${reward}_{S}$ and ${reward}_{T}$ are the reward values obtained from Eq. (6), $\mathrm{max}{Q}_{S}^{target}$ and $\mathrm{max}{Q}_{T}^{target}$ are the maximum $Q$ values of *target_net* in the current state, and ${Q}_{S}^{eval}$ and ${Q}_{T}^{eval}$ are the $Q$ values of *eval_net* in the current state.

Thus, the total loss function, $\mathcal{L}$, is obtained by summing the loss functions of Eqs. (3)–(5), (7), and (8). Parameter ${\theta }_{base}$ of this base-model can be trained and updated using the gradient descent method:

9 $$\mathcal{L}=({\mathcal{L}}_{S}^{1}+{\mathcal{L}}_{T}^{1})+{\mathcal{L}}_{M}^{2}+({\mathcal{L}}_{S}^{3}+{\mathcal{L}}_{T}^{3})$$

10 $${\theta }_{base}={\theta }_{base}-\alpha {\nabla }_{{\theta }_{base}}\mathcal{L}$$

where $\alpha$ is the learning rate of the base-model, and ${\nabla }_{{\theta }_{base}}$ is the gradient with respect to ${\theta }_{base}$.

### Meta-model learning

The meta-learning method derives the law of deformation control from multiple tasks (pairs) to obtain a meta-invariant feature space. This achieves the machining deformation control of different batches of blanks. The network structure of the meta-model is the same as that of the base-model, but the parameters are different. The meta-model can learn from different base-models and rapidly adapt to a new task with limited data. With the help of the meta-model memory, the historical experience of the base-models is stored for training and updating the meta-model parameters:

11 $${\theta }_{meta}={\theta }_{meta}-\beta {\nabla }_{{\theta }_{meta}}\sum_{{\mathcal{T}}_{i}\sim p(\mathcal{T})}{\mathcal{L}}_{{\mathcal{T}}_{i}}$$

where ${\theta }_{meta}$ is the meta-model parameter, $\beta$ is the meta-learning rate, ${\nabla }_{{\theta }_{meta}}$ is the gradient with respect to ${\theta }_{meta}$, ${\mathcal{T}}_{i}$ is *i*^th^ task, and $p\left(\mathcal{T}\right)$ is the task distribution set.

The algorithm of the reinforcement learning method based on the meta-invariant feature space is outlined as follows.

**Algorithm 1:**
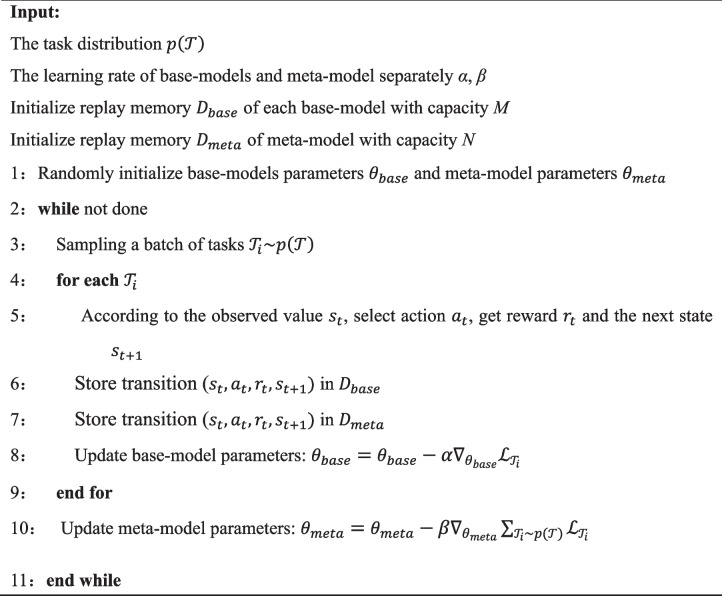
Reinforcement learning based on meta-invariant feature space

## Results and discussion

### Machining parameters and finite element settings

**Fig. 6:**
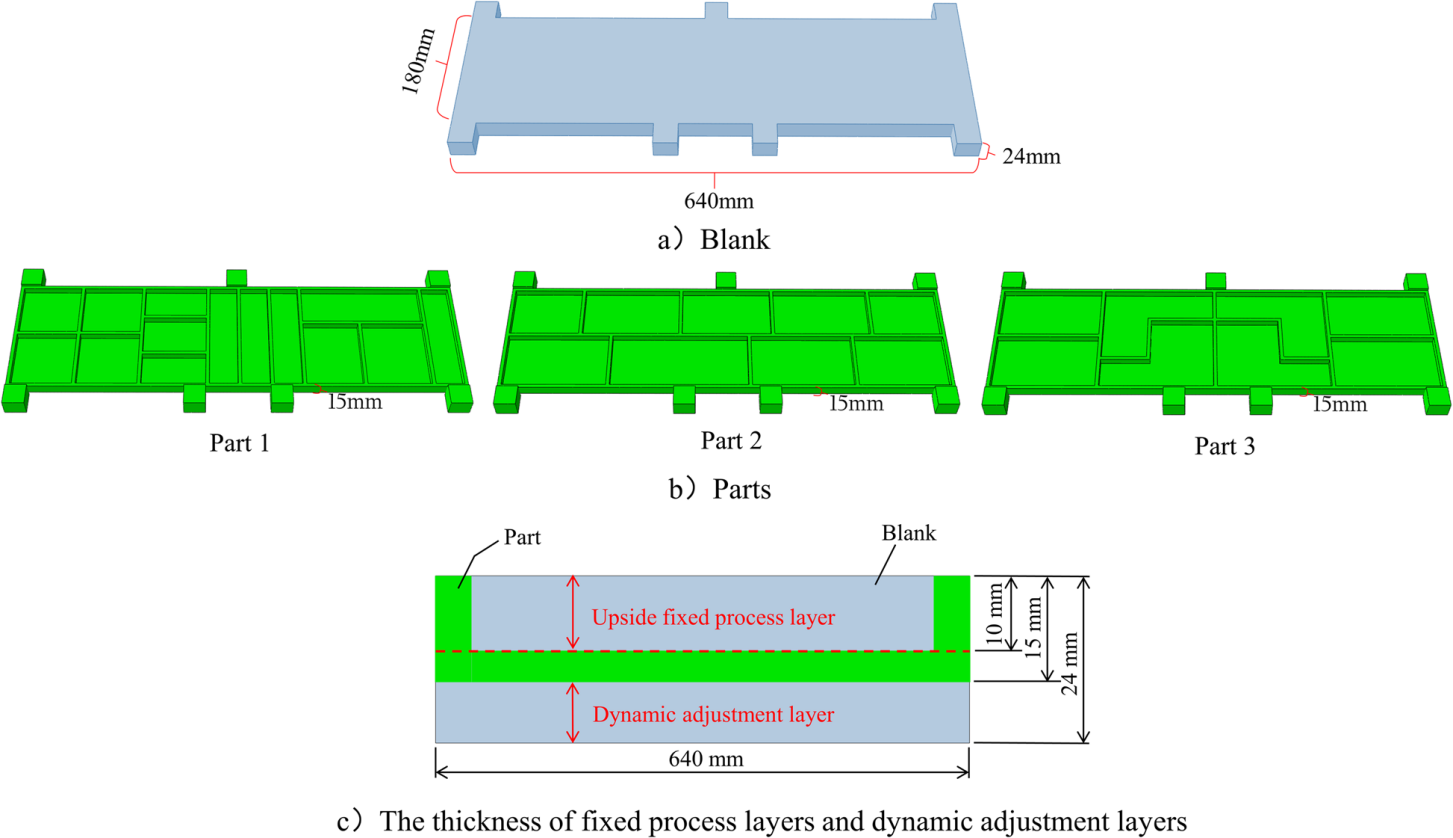
Schematic of blank and parts

In this study, the deformation of the machined part was controlled by changing its position in the blank in the thickness direction, as shown in Figs. 1 and 5. The shapes of the three analogous parts are shown in Fig. 6b. The blank dimensions were the same for the three parts (Fig. 6a), whose material was 7075-T651 aluminum alloy. The thicknesses of the fixed process and dynamic adjustment layers of the part were 10 and 9 mm, respectively (Fig. 6c).

**Fig. 7:**
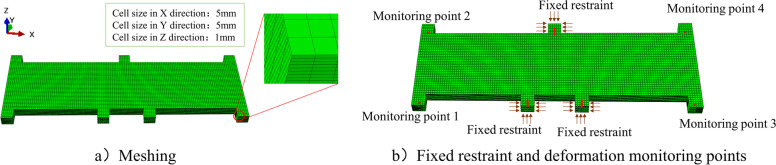
Machining parameters and finite element settings

The simulation was performed using ${ABAQUS}^{TM}$. The meshing of the finite elements is shown in Fig. 7a, and the fixed restraints are shown in Fig. 7b. Regarding data collection during machining, the deformation forces of the parts were probed at four monitoring points, as shown in Fig. 7b.

### Initial residual-stress distributions of different blanks

In this subsection, simulations of the initial residual-stress distributions of different blank batches for monitoring the data collection are discussed. Aluminum alloys are generally prepared by hot rolling, quenching, stretching, aging, and other steps [40]. During quenching, significant residual stress is generated, and the blank surface is under compressive stress, whereas the core is under tensile stress. A prestretching process is typically applied to reduce stress and induce 1%-3% plastic deformation to the blank on a stretching machine, thereby redistributing the residual stress in the thickness direction [41]. In this study, ${ABAQUS}^{TM}$ was used to simulate quenching and prestretching of the materials to obtain the resultant residual-stress distribution [42, 43].

The mechanical and thermophysical properties of 7075 aluminum alloy were obtained from ref. [44]. The specific preprocess is as follows. First, the material was heated to 465–475 °C. Next, it was subjected to quenching in water at 25 °C and mechanically stretched, exhibiting a 1%–3% permanent plastic deformation. In the simulations, six groups with different parameter combinations were selected, and each group corresponded to a machining condition (working condition or batch). The temperature and mechanical stretching parameters are listed in Table 1.

**Table 1:** Heating temperature and mechanical stretching parameters for residual stress simulation

	Group 1	Group 2	Group 3	Group 4	Group 5	Group 6
Heating temperature	465 °C	470 °C	475 °C	465 °C	475 °C	465 °C
Stretching rate	1%	2%	3%	3%	1%	2%

**Fig. 8:**
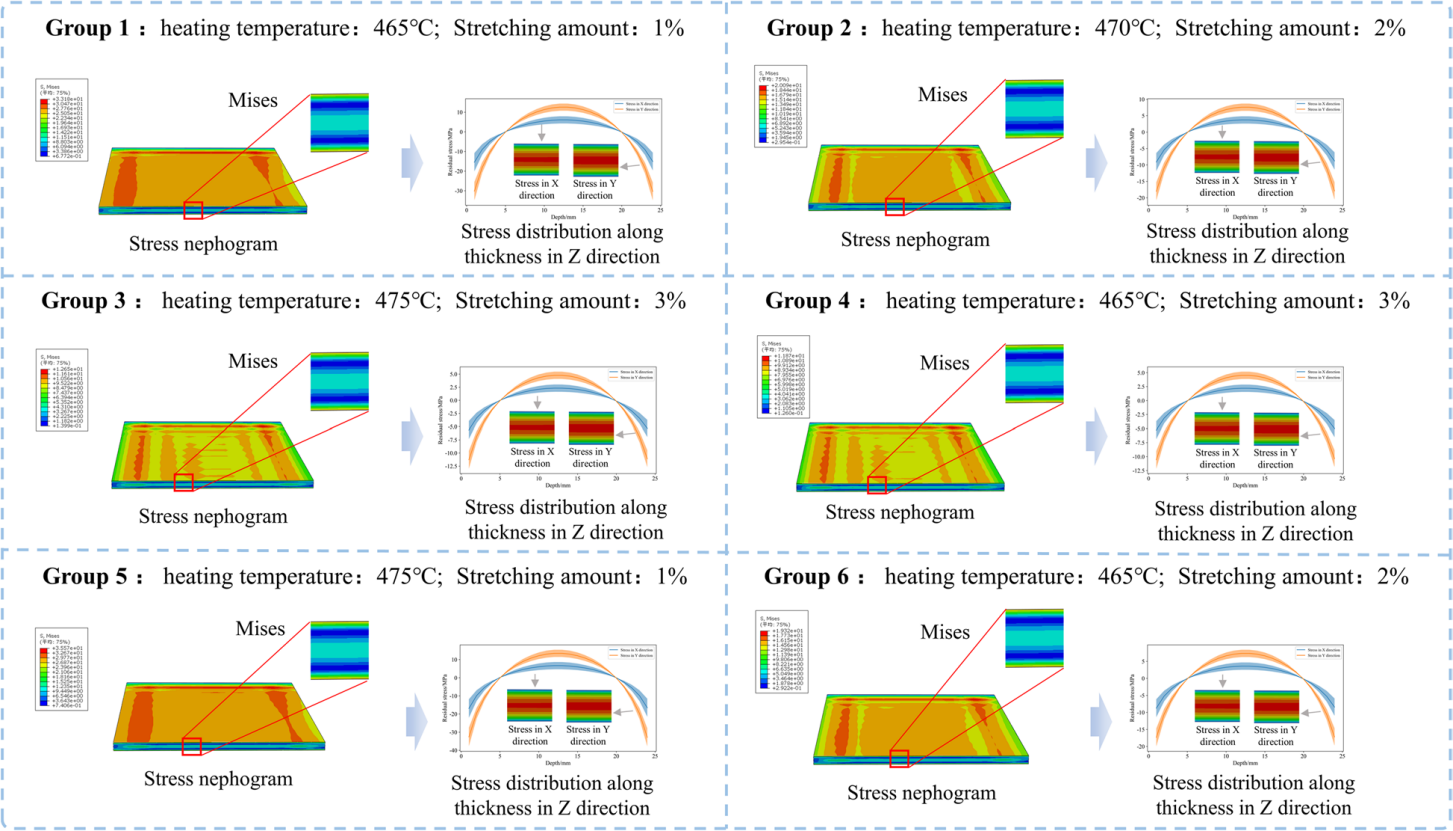
Preparation parameters and residual-stress distributions of six groups of 7075 aluminum alloy

Figure 8 shows the different residual stress fields of the six groups. Compressive stress existed near the blank surface, and tensile stress existed in the interior, conforming to the stress distribution. The six stress distribution groups were regarded as the stress field distributions of the six blank batches. However, the heating temperature and stretching amount varied within a specific range. Therefore, it was difficult to precisely control them at a constant value, which was a reason for the random difference in the stress field within the same batch. Assuming that this random error followed a normal distribution, the simulated stress distribution was adopted as mean $\mu$ in each group, and the field distributions were randomized using the standard deviation, $\sigma =10\%\times \mu$. Many subconditions were then randomly sampled within this batch (in this study, there were 200 samples per group).

### Machining condition pairing

For the implementation, Groups 1–5 were used to train the base-models and meta-model, whereas Group 6 was used for testing. For each group (batch), 200 stress distributions were sampled. In each sample, the fixed process layer was further divided into five sublayers, which were sequentially machined. When machining each sublayer, four force probes at the monitoring points received the deformation force (Fig. 7b). Each sample had 5 × 4 = 20 deformation forces, forming a vector *x* of length 20. Thus, each group contained 200 samples, forming a 200 × 20 input data matrix. Before model training, the machining conditions were paired using the MMD obtained from Eq. (2) based on the deformation force samples of the different parts, as listed in Table 2.

**Table 2:** Condition pairing of three test parts

Part 1	Condition pair	Group 1 **|** Group 5	Group 5 **|** Group 2	Group 2 **|** Group 3	Group 3 **|** Group 4
	MMD	0.900	6.257	5.562	0.335
Part 2	Condition pair	Group 1 **|** Group 5	Group 5 **|** Group 2	Group 2 **|** Group 3	Group 3 **|** Group 4
	MMD	0.850	6.168	5.575	0.306
Part 3	Condition pair	Group 1 **|** Group 5	Group 5 **|** Group 2	Group 2 **|** Group 3	Group 3 **|** Group 4
	MMD	0.820	6.111	5.499	0.289

### Model training

**Fig. 9:**
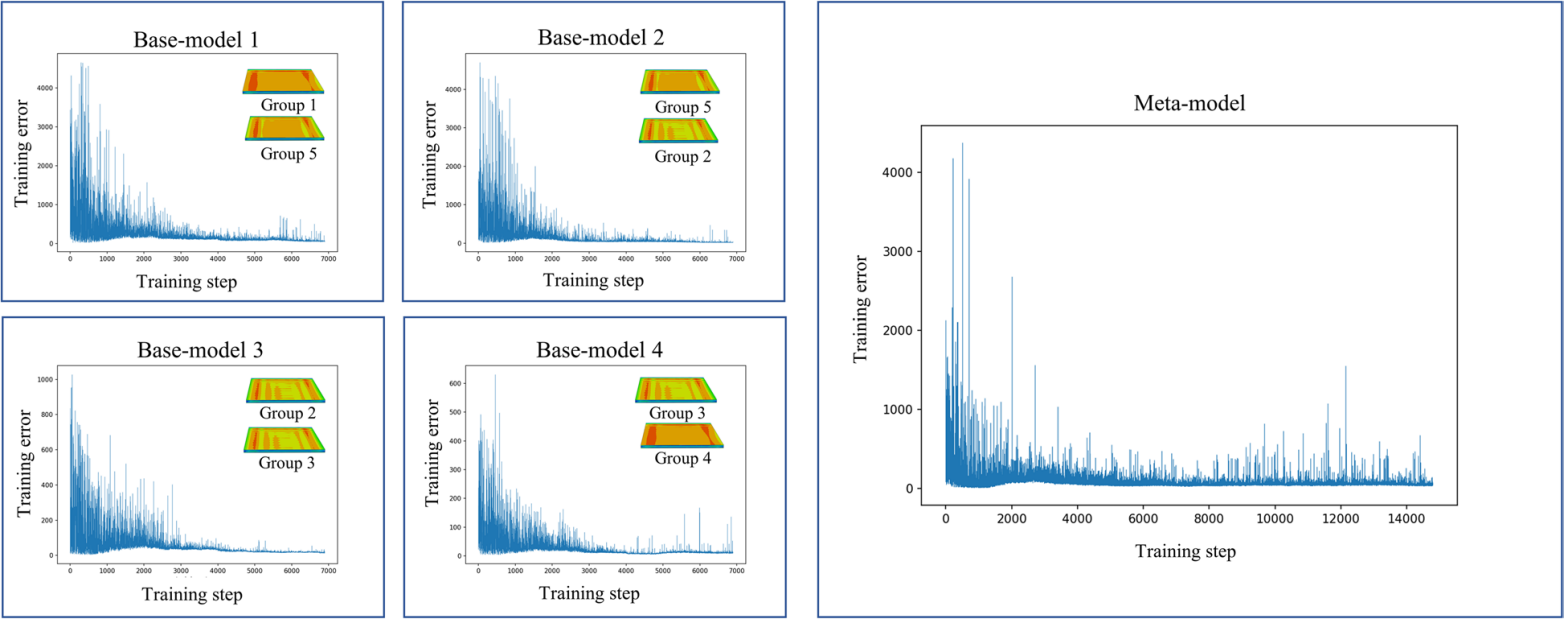
Convergence curves in training process of Part 1

**Fig. 10:**
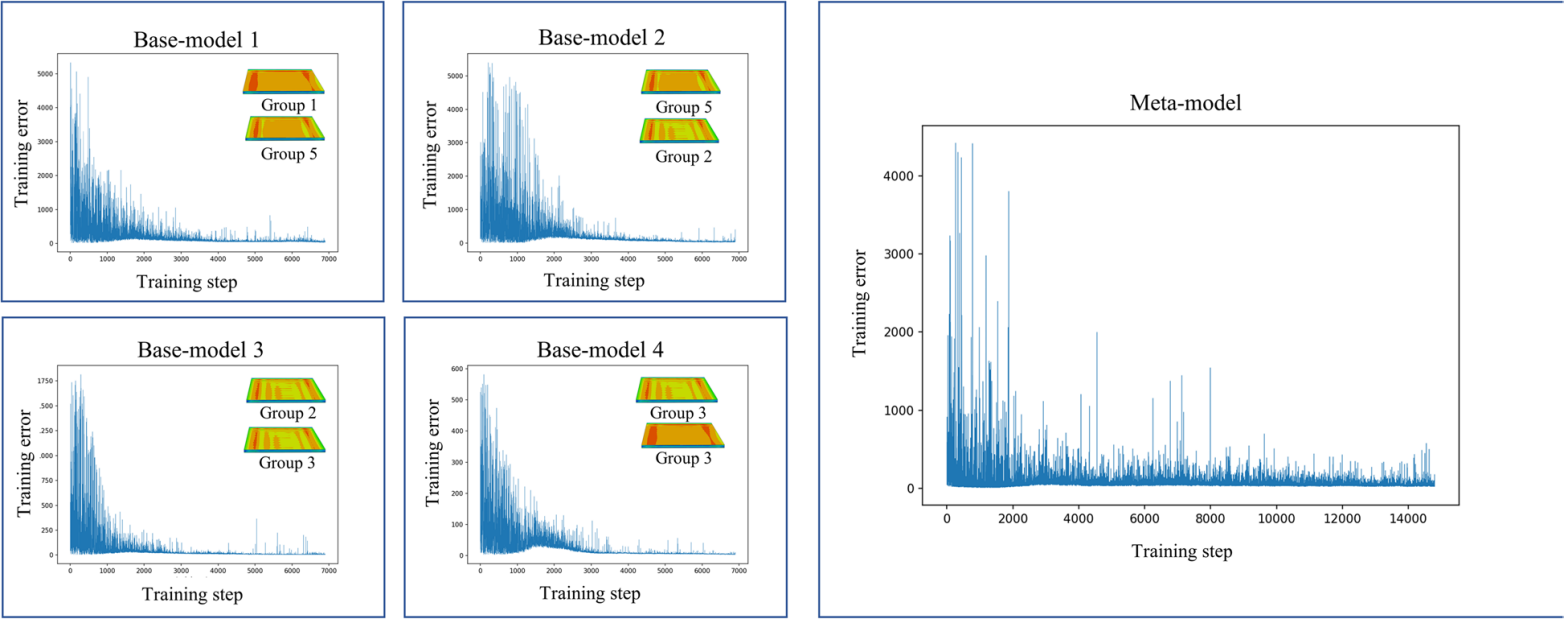
Convergence curves in training process of Part 2

**Fig. 11:**
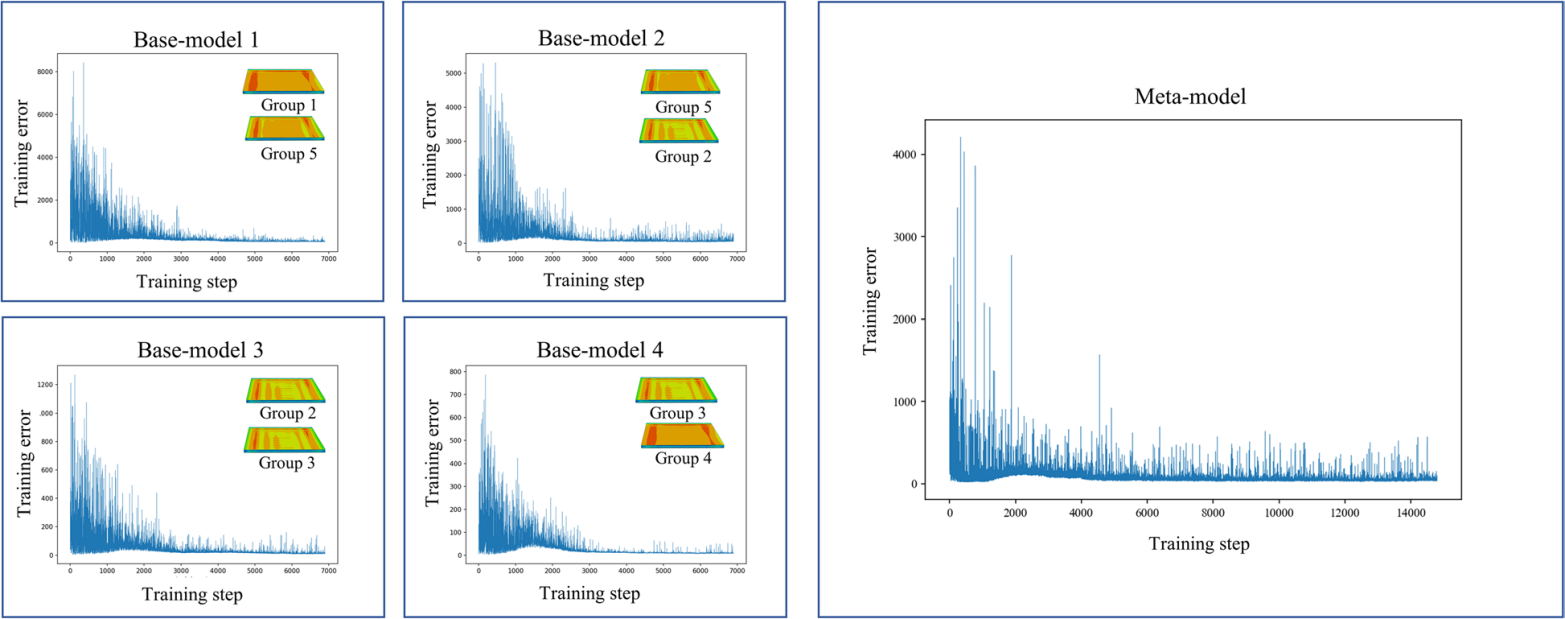
Convergence curves in training process of Part 3

The base-models and meta-model were trained according to the pairing results. The convergence curves of Parts 1, 2, and 3 plotted during the training are shown in Figs. 9, 10, and 11, respectively. For the base-model training, the training error sharply fluctuated at the beginning because the reinforcement learning used a greedy strategy to randomly select actions during the initial stage. With increased training steps, all the base-models in the four pairs learned how to make correct decisions from experience; therefore, the training errors gradually decreased and eventually stabilized. The meta-model learned from the experience of the base-models. Despite the fluctuating errors in the later stage of the training curve owing to significant differences among the conditions, the meta-model converged gradually. Verification of the final deformation control performance is presented in the next subsection. Additional training steps can be incorporated to reduce training loss and improve convergence results.

### Comparative verification and discussion

The proposed method is compared with the middle positioning method (that is, positioning the part in the middle position in the thickness direction) and the meta-reinforcement learning method [38] to verify the deformation control effect of the proposed method. We sampled 100 stress distributions from Group 6 as 100 testing samples. Similar to the training stage, each sample obtained 20 deformation forces after machining the fixed process layer. For each testing sample vector from Group 6, the MMDs between the sample and all input data matrices from other groups were first calculated, and the group with the minimum MMD was selected for pairing, for example, Group 3. Next, the base-model, which only tested the trained meta-model by assigning Groups 3 and 6 as Agent_S and Agent_T, respectively, was skipped. Finally, the meta-model could rapidly generalise to this new pair and make correct machining position decisions. The machining deformation of each testing sample was defined as the maximum absolute value of the machining deformations probed at the four monitoring points in Fig. 7c.

**Fig. 12:**
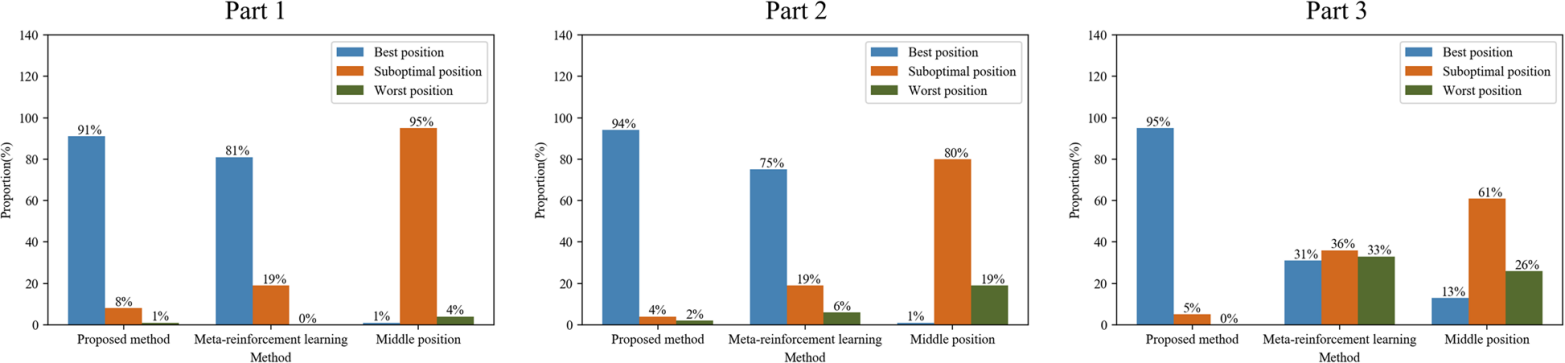
Deformation control effects of three methods on three test parts

The machining deformations of the 100 testing cases validated using these three algorithms are presented in the Appendix. These three methods are ranked as *best position*, *suboptimal position*, and *worst position*. The “best position,” “worst position,” and “sub-optimal position” indicate the smallest deformation, largest deformation, and somewhere in between, respectively. If two methods yield a similar deformation value, they rank the best or suboptimal position according to the value compared to the third value. The ranks of the 100 samples for each method are shown in Fig. 12.

Based on the results of the comparative experiments, the proposed algorithm performed best in all three parts. Taking Part 1 as an example, the decision-making results of the proposed method are 91% for the optimal position, 8% for the suboptimal position, and only 4% for the worst position. Compared with the other two methods, the decision-making results of the proposed meta-reinforcement learning method are 81% for the optimal position and 19% for the suboptimal position. In contrast, those of the middle position method are 1% for the optimal position, 95% for the suboptimal position, and 4% for the worst position. Similar conclusions can be drawn from Fig. 12 for the other two parts. From the controlling effect perspective, the proposed method not only exhibits the highest accuracy in controlling machining deformation but also shows good stability to the shape of a part. The meta-reinforcement learning method fares significant worse in the third part than in the other two parts. However, the proposed method achieves improved and stable performance in all three parts, demonstrating that the proposed method learns the intrinsic laws governing stress distributions and can make correct and stable decisions.

## Conclusions

This study proposes a reinforcement learning method based on a meta-invariant feature space used to control machining deformation with different batches of blanks. The proposed method first establishes two subnetworks to learn the invariant features of the paired conditions through cooperative learning. A meta-model is then used to learn the essential laws governing the spatial changes in invariant features under multiple pairs of conditions based on the meta-learning principle. The meta-model can be adapted to achieve precise machining deformation control under new conditions with only a small amount of monitoring data. Compared to two benchmarking methods, the proposed method achieves improved deformation control when a new batch of blanks is machined. Moreover, the proposed method can be valuable for solving other manufacturing problems caused by differences in task distribution.

In future studies, the efficiency of the model training should be considered. In addition, the proposed method was only verified in a simulation environment; although it is viable and practical, it must be validated based on physical machining experiments.

## Appendix

The machining deformation produced by different strategies under 100 samples of stress distribution

**Table:** 

Index	Part 1			Part 2			Part 3		
	Proposed method (mm)	Meta-reinforcement learning (mm)	Middle position (mm)	Proposed method (mm)	Meta-reinforcement learning (mm)	Middle position (mm)	Proposed method (mm)	Meta-reinforcement learning (mm)	Middle position (mm)
1	0.0239	0.0239	0.0405	0.0128	0.0128	0.0461	0.0336	0.0660	0.0336
2	0.0208	0.0208	0.0577	0.0148	0.0148	0.0501	0.0144	0.0316	0.0520
3	0.0192	0.0192	0.0580	0.0102	0.0102	0.0563	0.0132	0.0132	0.0513
4	0.0223	0.0223	0.0476	0.0159	0.0159	0.0697	0.0291	0.0678	0.0393
5	0.0213	0.0213	0.0441	0.0173	0.0173	0.0544	0.0281	0.0675	0.0369
6	0.0122	0.0122	0.0455	0.0238	0.0238	0.0520	0.0185	0.0373	0.0393
7	0.0127	0.0127	0.0473	0.0106	0.0331	0.0539	0.0193	0.0659	0.0409
8	0.0130	0.0130	0.0520	0.0260	0.0260	0.0276	0.0199	0.0573	0.0453
9	0.0240	0.0240	0.0459	0.0220	0.0220	0.0649	0.0301	0.0664	0.0386
10	0.0092	0.0092	0.0444	0.0229	0.0229	0.0482	0.0182	0.0542	0.0370
11	0.0220	0.0220	0.0591	0.0200	0.0200	0.0588	0.0351	0.0351	0.0514
12	0.0183	0.0183	0.0550	0.0124	0.0124	0.0481	0.0253	0.0603	0.0468
13	0.0111	0.0111	0.0495	0.0229	0.0229	0.0682	0.0139	0.0139	0.0433
14	0.0188	0.0188	0.0648	0.0134	0.0134	0.0428	0.0103	0.0579	0.0567
15	0.0140	0.0140	0.0458	0.0164	0.0164	0.0557	0.0114	0.0396	0.0400
16	0.0128	0.0128	0.0545	0.0085	0.0230	0.0489	0.0143	0.0421	0.0482
17	0.0165	0.0165	0.0639	0.0218	0.0218	0.0446	0.0121	0.0570	0.0557
18	0.0137	0.0137	0.0504	0.0132	0.0132	0.0664	0.0209	0.0550	0.0435
19	0.0127	0.0127	0.0525	0.0152	0.0461	0.0461	0.0205	0.0205	0.0450
20	0.0168	0.0168	0.0651	0.0122	0.0331	0.0461	0.0108	0.0505	0.0573
21	0.0352	0.0095	0.0352	0.0151	0.0151	0.0421	0.0169	0.0438	0.0305
22	0.0121	0.0121	0.0585	0.0151	0.0511	0.0570	0.0103	0.0103	0.0517
23	0.0149	0.0149	0.0431	0.0146	0.0146	0.0513	0.0209	0.0457	0.0371
24	0.0193	0.0604	0.0604	0.0258	0.0301	0.0258	0.0113	0.0113	0.0533
25	0.0111	0.0111	0.0515	0.0443	0.0151	0.0605	0.0138	0.0563	0.0440
26	0.0097	0.0097	0.0498	0.0148	0.0148	0.0601	0.0182	0.0182	0.0429
27	0.0136	0.0136	0.0581	0.0125	0.0125	0.0545	0.0104	0.0383	0.0508
28	0.0178	0.0580	0.0580	0.0196	0.0196	0.0488	0.0510	0.0543	0.0510
29	0.0122	0.0122	0.0573	0.0195	0.1160	0.0606	0.0189	0.0511	0.0501
30	0.0163	0.0163	0.0557	0.0131	0.0131	0.0529	0.0242	0.0242	0.0474
31	0.0146	0.0146	0.0600	0.0197	0.0197	0.0600	0.0524	0.0606	0.0524
32	0.0129	0.0129	0.0423	0.0125	0.0317	0.0675	0.0199	0.0503	0.0356
33	0.0195	0.0195	0.0487	0.0132	0.0132	0.0478	0.0262	0.0262	0.0411
34	0.0150	0.0150	0.0570	0.0119	0.0293	0.0568	0.0105	0.0105	0.0500
35	0.0253	0.0296	0.0253	0.0138	0.0138	0.0451	0.0196	0.0587	0.0196
36	0.0150	0.0449	0.0449	0.0185	0.0185	0.0568	0.0227	0.0227	0.0380
37	0.0150	0.0460	0.0460	0.0096	0.0283	0.0611	0.0598	0.0388	0.0388
38	0.0674	0.0124	0.0674	0.0265	0.0265	0.0785	0.0157	0.0410	0.0591
39	0.0243	0.0353	0.0590	0.0141	0.0432	0.0577	0.0282	0.0345	0.0526
40	0.0215	0.0215	0.0645	0.0208	0.0208	0.0482	0.0154	0.0381	0.0571
41	0.0394	0.0194	0.0597	0.0182	0.1061	0.0690	0.0123	0.0499	0.0524
42	0.0130	0.0130	0.0661	0.0151	0.0470	0.0570	0.0130	0.0518	0.0579
43	0.0154	0.0154	0.0550	0.0098	0.0098	0.0498	0.0225	0.0643	0.0469
44	0.0547	0.0146	0.0499	0.0117	0.0472	0.0522	0.0217	0.0628	0.0428
45	0.0129	0.0129	0.0443	0.0223	0.0223	0.0594	0.0206	0.0562	0.0372
46	0.0290	0.0290	0.0358	0.0095	0.0095	0.0533	0.0293	0.0342	0.0293
47	0.0176	0.0176	0.0558	0.0310	0.0310	0.0691	0.0257	0.0257	0.0470
48	0.0082	0.0486	0.0486	0.0143	0.0143	0.0588	0.0176	0.0176	0.0437
49	0.0142	0.0634	0.0634	0.0244	0.0354	0.0591	0.0132	0.0132	0.0549
50	0.0130	0.0130	0.0493	0.0539	0.0170	0.0533	0.0614	0.0614	0.0421
51	0.0442	0.0260	0.0442	0.0447	0.0290	0.0358	0.0329	0.0329	0.0368
52	0.0194	0.0194	0.0508	0.0103	0.0103	0.0360	0.0137	0.0330	0.0455
53	0.0202	0.0271	0.0271	0.0195	0.0195	0.0655	0.0221	0.0269	0.0221
54	0.0124	0.0124	0.0481	0.0146	0.0146	0.0465	0.0200	0.0482	0.0415
55	0.0148	0.0567	0.0567	0.0263	0.0263	0.0447	0.0150	0.0581	0.0494
56	0.0124	0.0124	0.0581	0.0189	0.0189	0.0555	0.0188	0.0444	0.0512
57	0.0306	0.0306	0.0687	0.0590	0.0346	0.0590	0.0617	0.0457	0.0617
58	0.0111	0.0111	0.0637	0.0132	0.0132	0.0506	0.0154	0.0504	0.0560
59	0.0290	0.0115	0.0564	0.0136	0.1143	0.0530	0.0155	0.0381	0.0505
60	0.0236	0.0284	0.0284	0.0152	0.0152	0.0571	0.0229	0.0562	0.0229
61	0.0260	0.0260	0.0443	0.0157	0.0157	0.0553	0.0334	0.0703	0.0371
62	0.0232	0.0232	0.0539	0.0266	0.0266	0.0454	0.0305	0.0305	0.0446
63	0.0183	0.0183	0.0602	0.0154	0.0154	0.0436	0.0131	0.0131	0.0523
64	0.0088	0.0493	0.0493	0.0598	0.0232	0.0598	0.0172	0.0172	0.0422
65	0.0097	0.0097	0.0614	0.0169	0.0169	0.0643	0.0167	0.0604	0.0540
66	0.0225	0.0594	0.0594	0.0231	0.0979	0.0600	0.0157	0.0157	0.0523
67	0.0146	0.0146	0.0600	0.0130	0.0130	0.0594	0.0096	0.0515	0.0535
68	0.0264	0.0452	0.0452	0.0130	0.0339	0.0547	0.0374	0.0340	0.0374
69	0.0099	0.0099	0.0532	0.0094	0.0498	0.0647	0.0165	0.0421	0.0458
70	0.0230	0.0230	0.0596	0.0241	0.0241	0.0548	0.0160	0.0453	0.0524
71	0.0126	0.0126	0.0500	0.0209	0.0209	0.0579	0.0131	0.0131	0.0435
72	0.0220	0.0220	0.0672	0.0124	0.0124	0.0581	0.0157	0.0515	0.0587
73	0.0346	0.0236	0.0708	0.0246	0.0246	0.0717	0.0169	0.0450	0.0638
74	0.0258	0.0274	0.0274	0.0123	0.0123	0.0574	0.0312	0.0546	0.0217
75	0.0092	0.0092	0.0530	0.0098	0.0480	0.0503	0.0457	0.0588	0.0457
76	0.0163	0.0163	0.0554	0.0118	0.0118	0.0645	0.0230	0.0561	0.0474
77	0.0081	0.0081	0.0570	0.0211	0.0211	0.0280	0.0164	0.0164	0.0489
78	0.0235	0.0235	0.0518	0.0260	0.0260	0.0442	0.0292	0.0622	0.0443
79	0.0602	0.0087	0.0602	0.0244	0.0244	0.0292	0.0143	0.0373	0.0528
80	0.0270	0.0270	0.0684	0.0275	0.0275	0.0689	0.0204	0.0457	0.0610
81	0.0101	0.0101	0.0562	0.0089	0.0089	0.0555	0.0190	0.0618	0.0475
82	0.0320	0.0320	0.0401	0.0431	0.0431	0.0660	0.0323	0.0323	0.0323
83	0.0260	0.0260	0.0781	0.0184	0.0451	0.0585	0.0184	0.0609	0.0696
84	0.0116	0.0116	0.0455	0.0084	0.0527	0.0499	0.0183	0.0420	0.0389
85	0.0150	0.0587	0.0587	0.0134	0.0134	0.0524	0.0429	0.0429	0.0514
86	0.0204	0.0204	0.0479	0.0165	0.0165	0.0556	0.0262	0.0605	0.0410
87	0.0088	0.0554	0.0554	0.0203	0.0203	0.0517	0.0152	0.0474	0.0492
88	0.0199	0.0199	0.0703	0.0157	0.0766	0.0456	0.0111	0.0620	0.0622
89	0.0181	0.0689	0.0689	0.0084	0.0084	0.0574	0.0105	0.0105	0.0610
90	0.0156	0.0156	0.0695	0.0244	0.0244	0.0463	0.0091	0.0091	0.0624
91	0.0137	0.0137	0.0573	0.0148	0.0591	0.0640	0.0125	0.0524	0.0503
92	0.0161	0.0161	0.0524	0.0329	0.0329	0.0410	0.0244	0.0623	0.0446
93	0.0268	0.0268	0.0349	0.0205	0.0205	0.0709	0.0277	0.0342	0.0277
94	0.0149	0.0149	0.0568	0.0132	0.0132	0.0495	0.0124	0.0568	0.0487
95	0.0148	0.0148	0.0417	0.0188	0.0188	0.0606	0.0221	0.0221	0.0349
96	0.0164	0.0164	0.0535	0.0521	0.0099	0.0616	0.0234	0.0508	0.0459
97	0.0087	0.0087	0.0640	0.0118	0.0118	0.0501	0.0171	0.0577	0.0555
98	0.0541	0.0122	0.0541	0.0277	0.0277	0.0358	0.0176	0.0397	0.0481
99	0.0127	0.0127	0.0521	0.0094	0.0438	0.0446	0.0203	0.0203	0.0448
100	0.0079	0.0495	0.0495	0.0245	0.0245	0.0411	0.0159	0.0159	0.0419

## Data Availability

The datasets used and/or analysed during the current study are available from the corresponding author on reasonable request.

## Abbreviations

MMD: Maximum mean discrepancy
DQN: Deep Q-network
MSE: Mean square error
